# Immune-related miRNA signature identifies prognosis and immune landscape in head and neck squamous cell carcinomas

**DOI:** 10.1042/BSR20201820

**Published:** 2020-11-17

**Authors:** Bo Ma, Hui Li, Jia Qiao, Tao Meng, Riyue Yu

**Affiliations:** 1 Department of Stomatology, Beijing Shijitan Hospital, Capital Medical University, Beijing 100038, China; 2 Department of Stomatology, Hui Ya Hospital of The First Affiliated Hospital, Sun Yat-Sen University, Huizhou 516080, China

**Keywords:** bioinformatics, cancer, immunology, microRNA

## Abstract

**Background:** Head and neck squamous cell carcinoma (HNSCC) is recognised as an immune active cancer, but little is known about the role of microRNAs (miRNAs) in it. In the present study, we aim to determine a prognostic and immune-related miRNAs signature (IRMS) in HNSCC.

**Methods:** Spearman correlation analysis was used to screen out prognostic immune-related miRNAs based on single-sample gene set enrichment analysis (ssGSEA). Least absolute shrinkage and selection operator (LASSO) Cox regression model was used to establish IRMS in HNSCC. Then, the influence of the IRMS on HNSCC was comprehensively analysed.

**Results:** We obtained 11 prognostic immune-related miRNAs based on ssGSEA. Then an IRMS integrated with six miRNAs was established through LASSO Cox regression analysis. The stratification survival analysis indicated that IRMS was independent from other characteristics and performed favourably in the overall survival (OS) prediction. The function annotation suggested that IRMS was highly associated with the immune-related response biological processes and pathways which are so important for tumorigenesis of HNSCC. Moreover, the nomogram demonstrated that our model was identified as an independent prognostic factor. In addition, we found that IRMS was significantly correlated with the immune infiltration and expression of critical immune checkpoints, indicating that the poor prognosis might be caused partly by immunosuppressive microenvironment.

**Conclusion:** We established a novel IRMS, which exhibited a potent prognostic value and could be representative of immune status in HNSCC.

## Background

Head and neck squamous cell carcinoma (HNSCC) is a heterogeneous solid malignancy which originates from distinct sites of mucosal linings of the upper aerodigestive tract, including the oral cavity, buccal mucosa, tongue, pharynx and larynx, which has nearly 650000 new cases and 350000 deaths and ranks as the sixth most common cancer worldwide in 2018 [1]. Although combination of the surgery, chemotherapy and radiotherapy have transformed HNSCC from an incurable disease to a potentially curable one, nevertheless, the 5-year survival rate of patients with HNSCC is still less than 50% [2,3]. The classical known environmental risk factors for HNSCC are excessive tobacco and alcohol exposure [4,5]. Recently, infection with high-risk human papillomaviruses (HPVs) is known to be strongly associated with the development and prognosis of HNSCC, which have profoundly changed our knowledge of characteristics of the disease [6,7].

HNSCC is identified as an immunologically active tumour with higher immune infiltration among all types of cancers, which is also called ‘hot’ tumour [8]. In addition, HNSCC was characterized with moderate-high tumour mutational burden (TMB). But some clinical trials such as ‘CheckMate 141’ and ‘KEYNOTE-014’ indicated that only 15–20% of patients with platinum-refractory recurrent or metastatic HNSCC did respond to immunotherapy with programmed death-1 (PD-1)/programmed death-ligand-1 (PD-L1) immune-checkpoint inhibitors (ICIs) [9–12]. Recently, many factors have been reported to be associated with immunotherapy response as follows: the expression of immune checkpoints such as PD-L1 measured by immunohistochemistry (IHC), immune cells infiltration populations such as levels of CD8 T cells, and an ‘inflamed’ tumour phenotype established by IFNγ signature. Due to no uniform standards of above detection, there is currently no evidence for accurate biomarkers to predict response to ICIs in HNSCC [13–15]. Considering that, characterization of the tumour immune microenvironment (TIME), containing a great diversity of immune cells, which are collaborating with each other to generate a chronic inflammatory, immunosuppressive, and pro-angiogenic intratumoural atmosphere [16], is an urgent need and becoming more prominent in HNSCC.

Due to the development of transcriptome sequencing over past decades, we have found that nearly 70% of the genome is transcribed into RNA, and the majority of them are non-coding RNAs (ncRNAs) [17,18]. Among them, microRNAs (miRNAs), a class of smallest ncRNAs with a length of 19–25 nucleotides, have been reported to act as a regulator in variety of biological processes in eukaryotes [16,19]. MiRNAs exert their function by controlling the gene expression through post-translational modification via base-pairing mechanisms of silencing the 3′-untranslated region (UTR) district of target genes, which were validated in many studies [20–22]. Although recognized as ‘rubbish’ genes when first discovered [23], miRNAs have now been found to play a vital role in the tumorigenesis of many cancers, such as breast cancer [24], lung cancer [25], bladder cancer [26], prostate cancer [27] and so on. Furthermore, different literatures have obtained contradictory results of the relationship between the expression of miRNA and progression and clinical outcome of HNSCC [28]. Thus, recently some studies even evaluated the diagnostic or prognostic performance of miRNAs [29]. However, little is known about the relationship between the miRNAs and immune response in HNSCC.

In the present study, we aimed to characterize a comprehensive landscape of TIME and established a miRNAs signature which exhibited a high correlation with immune infiltration in HNSCC. Through Least absolute shrinkage and selection operator (LASSO) Cox regression analyses, we have found six prognostic immune-related miRNAs (miR-146a, miR-3127, miR-3913-2, miR-487b, miR-548k and miR-5690) and construct a model called immune-related miRNAs signature (IRMS) which could appropriately stratify the patients into low- and high-risk groups with distinct overall survival (OS). Furthermore, the function analyses and TIME landscape also demonstrated that IRMS was highly associated with the immune-related response biological processes and pathways which are so important for tumorigenesis of HNSCC. Moreover, the results showed that the IRMS was involved with the regulation of immune infiltration and immune checkpoints. In summary, we have established a novel IRMS, which exhibited a potent prognostic value and could be the representative of immune status in HNSCC.

## Materials and methods

### Data collection and processing

The publically available data of HTSeq-Fragments Per Kilobase per Million (FPKM), miRNA-seq and clinical information for HNSCC from the Cancer Genome Atlas (TCGA) was downloaded from the UCSC Xena (GDC hub) (https://tcga.xenahubs.net). The HTSeq-FPKM data were transferred to the transcripts per million reads (TPMs) which will represent as the expression of RNA at the highest expression according to the gene symbol in TCGA-HNSCC cohort. Moreover, the miRNA-seq data were recorded as the reads per million miRNAs mapped (RPMs) values. Then all data were log2-transformed for the subsequent analysis. After screening out the samples without OS, we got a total of 494 tumour samples both including HTSeq-FPKM and miRNA-seq data as an entire TCGA-HNSCC cohort, and then they were randomly divided into training and testing sets at cutoff 5:5. Data were analysed with the R (version 3.5.3) and R Bioconductor packages.

### Identification of immune-related miRNAs

The gene sets M13664 (immune system process) and M19817 (immune response) were used to representative of immune scores as previously described, which were obtained from Molecular Signatures Database (MSigDB) of Broad Institute (http://software.broadinstitute.org/gsea/index.jsp) [30,31]. The immune scores of each sample in TCGA-HNSCC cohort were measured with single-sample gene set enrichment analysis (ssGSEA) [32,33]. The low-expression miRNAs with row means = 0 were screened out from the further study. Then the Spearman correlation analysis was used to select immune-related miRNAs which were correlated with the immune scores (|R| > 0.3, *P*<0.05). After merging with the prognostic miRNAs obtained from univariate Cox regression analysis, the remaining miRNAs were identified as the prognostic immune-related candidate miRNAs. The process of the selection was shown in Table 1.

**Table 1:** The selection of immune related and prognostic candidate miRNAs

miRNA	Correlation (Spearman)	*P*-value (cor)	HR	HR.95L	HR.95H	*P*-value (unicox)
**miR-146a**	0.644524702	2.46E-59	0.772653243	0.681359	0.876179	5.81E-05
**miR-487b**	−0.360387828	1.35E-16	1.271831548	1.118144	1.446644	0.000252797
**miR-548k**	−0.376677378	4.23E-18	1.242971476	1.098107	1.406946	0.000581231
**miR-376c**	−0.310704718	1.62E-12	1.222860614	1.085092	1.37812	0.000970043
**miR-3913-2**	−0.356525566	2.98E-16	0.801301033	0.694831	0.924086	0.002324259
**miR-5690**	−0.304278027	4.84E-12	1.257939358	1.065295	1.485421	0.006814436
**miR-205**	0.305775339	3.76E-12	0.852731073	0.752777	0.965957	0.012263846
**miR-1304**	0.577360969	2.97E-45	1.100652549	1.01327	1.195571	0.023068045
**miR-296**	0.327245358	8.56E-14	1.103327359	1.007565	1.208192	0.033783171
**miR-4714**	0.415860758	4.43E-22	1.188873215	1.008172	1.401963	0.039713813
**miR-181c**	−0.351469885	8.26E-16	0.82607009	0.686433	0.994113	0.043129295
miR-523	−0.32088081	2.71E-13	1.202925994	0.985622	1.468139	0.069140312
miR-106a	0.377274673	3.71E-18	0.917459266	0.830629	1.013366	0.089465871
miR-361	−0.310823191	1.59E-12	0.819981972	0.639873	1.050788	0.116772429
miR-129-1	0.507641325	1.02E-33	1.056784396	0.981658	1.13766	0.142120519
miR-326	0.497954346	2.58E-32	1.096903098	0.96943	1.241138	0.142269517
miR-6879	0.315674394	6.82E-13	0.838292082	0.658545	1.0671	0.151995152
miR-193b	0.359800501	1.52E-16	1.091452357	0.964624	1.234956	0.164986334
miR-9-1	−0.418543759	2.26E-22	0.961275063	0.908705	1.016887	0.168702849
miR-653	0.5535918	5.17E-41	0.934983492	0.842994	1.037011	0.203299606
miR-548j	0.489840013	3.56E-31	0.906207943	0.776754	1.057236	0.210473388
miR-1245a	0.317381693	5.05E-13	1.073503023	0.959439	1.201127	0.215894592
miR-4473	−0.437739522	1.53E-24	0.907462199	0.773637	1.064436	0.232925168
miR-501	0.305381876	4.02E-12	0.908187928	0.774179	1.065394	0.237087127
miR-675	0.408394778	2.80E-21	1.037506599	0.974924	1.104107	0.24607702
miR-6737	0.324985472	1.29E-13	1.12486541	0.920959	1.373918	0.248885676
miR-940	0.47804624	1.43E-29	1.089720355	0.938344	1.265518	0.260172886
miR-550a-3	0.363164519	7.58E-17	1.086452566	0.930047	1.269161	0.295776626
miR-196a-2	0.42859496	1.72E-23	1.035014567	0.970237	1.104117	0.296636977
miR-221	0.404001495	8.09E-21	0.923679504	0.783251	1.089285	0.345401615
hsa-let-7a-3	−0.393225369	1.02E-19	0.92035581	0.772324	1.09676	0.353594112
miR-4536-2	0.345801389	2.54E-15	1.110155192	0.883875	1.394365	0.368889499
miR-4661	−0.370262118	1.69E-17	1.059642919	0.922974	1.21655	0.410922675
miR-585	−0.345006349	2.97E-15	0.951607609	0.840237	1.077739	0.434757913
miR-4638	−0.368646987	2.39E-17	0.95330424	0.833598	1.0902	0.48485908
miR-30a	0.423001428	7.29E-23	1.045150964	0.915027	1.193779	0.515067192
miR-4742	0.389659954	2.33E-19	0.954760867	0.817322	1.115311	0.559370397
miR-216b	−0.305057518	4.24E-12	1.06367971	0.850033	1.331024	0.589439038
miR-6805	0.577734346	2.53E-45	1.060185929	0.852369	1.318671	0.599569784
miR-3926-2	0.385359389	6.17E-19	0.96179816	0.830837	1.113403	0.601975079
miR-194-2	0.338954484	9.56E-15	0.958995856	0.81817	1.124061	0.60536411
miR-514a-2	0.464984652	7.21E-28	1.015569685	0.957086	1.077627	0.60967325
miR-5092	0.479340234	9.58E-30	1.042248581	0.888606	1.222457	0.611068945
miR-423	−0.401046939	1.64E-20	0.934416479	0.705272	1.238011	0.636525413
miR-324	−0.360491853	1.32E-16	0.956517202	0.783613	1.167573	0.66210239
miR-1180	−0.321356109	2.49E-13	1.024439504	0.904881	1.159795	0.702942983
miR-3065	0.34511512	2.90E-15	1.015293726	0.923638	1.116045	0.753202936
miR-519a-1	0.445486605	1.86E-25	1.012569994	0.936217	1.09515	0.754823773
miR-5008	0.301749926	7.38E-12	1.021218373	0.888865	1.17328	0.766870055
miR-4668	0.339986765	7.84E-15	1.015008301	0.901137	1.143268	0.806173007
miR-6892	0.652081158	3.80E-61	1.017135395	0.887557	1.165632	0.806949022
miR-1468	−0.302645507	6.36E-12	1.011910398	0.880769	1.162578	0.867219888
miR-3680-1	−0.341987082	5.33E-15	0.986498614	0.838084	1.161196	0.870198184
miR-1307	0.329937	5.22E-14	0.985398439	0.82334	1.179354	0.872524531
miR-508	0.444479641	2.45E-25	0.998533372	0.944367	1.055807	0.958865107
miR-6798	−0.423314656	6.73E-23	0.996856983	0.837694	1.186261	0.971706092
miR-6891	0.357738183	2.32E-16	0.997911147	0.87834	1.133759	0.974383332
miR-4763	0.456791647	7.77E-27	0.998139189	0.813824	1.224198	0.985733264

Abbreviation: HR, hazard ratio.

### Establishment and validation of prognostic IRMS

LASSO Cox regression analysis based on package ‘*glmnet*’ in R was utilized to build an immune-related miRNAs optimal prognostic signature (IRMS) for HNSCC based on selected prognostic immune-related candidate miRNAs mentioned above [34]. The formula for $\text{IRMS}\;\text{risk-score}=\sum_{i=1}^{n}\;({\text{coef}}_{\text{i}}\times {\text{Expr}}_{\text{i}})$. The Expr_i_ is the relative expression of miRNAs in the signature for patient i and coef_i_ is the LASSO Cox coefficient of the miRNA i in TCGA-HNSCC cohort. Furthermore, all patients were divided into low- and high-risk groups at the median cut-off according to the IRMS risk-score. The difference of OS between the IRMS high-/low-risk patients and distinct stratified clinicopathological characteristics were evaluated with Kaplan–Meier (KM) survival curves by using package ‘*survminer*’ in R. The prediction accuracy and ability of IRMS was assessed by time-dependent receiver operating characteristic curve (ROC), and the area under curve (AUC) for 1-, 3- and 5-year OS was measured by using package ‘*survivalROC*’ in R [35].

### Correlation between IRMS with other clinicopathological characteristics

The correlation between IRMS risk-scores with corresponding clinicopathological characteristics, including age, gender, grade, lympho-nodes positive by Hematoxylin and Eosin (HE), lymphovascular invasion status, margin status, pathological T stage, pathological N stage, pathological tumour node metastasis (TNM) stage, neoplasm cancer status, pathological extracapsular spread, primary therapy outcome and follow-up treatment outcome, was measured by *t* test or one-way ANOVA test and shown by violin plot. Furthermore, the correlation between clinicopathological characteristics with IRMS risk-level was also calculated by *χ*^2^ test and shown in cluster heat map. *P<0.05, **P<0.01, ***P<0.001.

### Functional and annotation analyses

The Hallmark gene sets, which consisted of 50 independent gene sets, were also downloaded from the MSigDB database of Broad Institute (http://software.broadinstitute.org/gsea/index.jsp) [36]. Gene Set Variation Analysis (GSVA) was used to analyse the enrichment of biological process and pathways due to IRMS risk-level through package ‘*GSVA*’ in R [37]. The cut-off of the significantly enriched pathways in Hallmark gene sets were identified as *P*<0.05 and *t* value > 2. Furthermore, ssGSEA score of each gene set in Hallmark gene sets were also measured in each sample in HNSCC cohort. The Spearman correlation analysis was used to detect the correlation between the IRMS risk-scores and immune-related candidate miRNAs.

### Establishment of a predictive nomogram

The univariate and multivariate Cox regression analyses were utilized to find independent prognostic factors by merging IRMS and other clinical features, which was then visualised via package ‘*forestplot*’ in R. The selected independent prognostic factors were integrated to establish nomogram through package ‘*rms*’, ‘*nomogramEx*’ and ‘*regplot*’ in R [38]. Furthermore, decision curve analysis (DCA) and calibration curves were used to see the reliability of our nomogram.

### The landscape of immune infiltration in HNSCC cohort

The gene set which was representative of different immune cell types was obtained from Bindea et al. [39]. Then levels of different immune cell types were calculated through ssGSEA based on expression of reference gene within the gene sets from RNA-seq data. We enrolled 24 types of immune cells in our study, including innate immune cells (dendritic cells [DCs], immature DCs [iDCs], activated DCs [aDCs], plasmacytoid DC [pDCs], eosinophils, mast cells, macrophages, natural killer cells [NKs], NK CD56dim cells, NK CD56bright cells and neutrophils) and adaptive immune cells (B cells, T cells, T helper cells, T helper 1 [Th1], Th2, T follicular helper gamma delta [Tγδ], CD8^+^ T, T central memory [Tcm], T effector memory [Tem], T follicular helper [Tfh] cells, T helper 17 (Th17) cells, regulatory T (Treg) cells and cytotoxic cells). The survival benefit of each immune cell was measured by KM survival analysis, and correlation between each immune cells and IRMS was measured by Spearman correlation analysis.

### Statistical analyses

Statistical significance for parameters between two groups or more than two groups was estimated by unpaired Student’s *t* test or one-way ANOVA test, respectively. The *χ^2^* test was applied to analyse the correlation between IRMS risk-level and clinical characteristics. Differences for survival benefits between different groups were assessed by KM survival curves by using the package ‘*survminer*’ in R. The correlation between two variables was measured by Spearman and distance correlation analyses. The independent prognostic factors were calculated and identified by univariate and multivariate cox proportional-hazard models. Nomogram, calibration curve and DCA were established due to Iasonos et al.’s suggestion [38]. The time-dependent ROC analyses were used to measure the predictive accuracy. All statistical analyses were performed with R software 3.5.3. Statistical significance was set at probability values of *P*<0.05.

## Results

### Identification of prognostic and immune-related miRNAs

**Figure 1:**
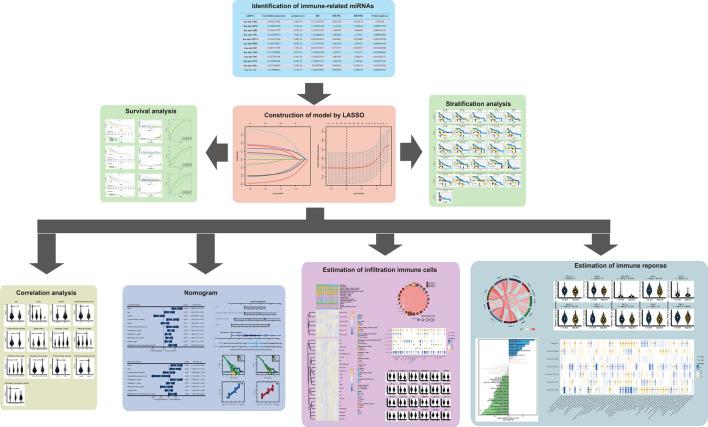
A study design and flow diagram

A study design and flow diagram can be found in Figure 1. We identified 494 samples with data of OS, HTSeq-FPKM and miRNA-seq as an entire TCGA-HNSCC cohort. After screening out the low expression miRNAs, we got 1881 miRNAs for further study. Furthermore, immune scores based on the M13664 (immune system process) and M19817 (immune response) gene sets were achieved through ssGSEA in each sample in TCGA-HNSCC cohort. Then 58 miRNAs with a correlation of |R| > 0.3 and *P*<0.05 were enrolled as the immune-related miRNAs via Spearman correlation analyses. Then 58 immune-related miRNAs were submitted for univariate Cox regression analysis. Finally, 11 prognostic immune-related candidate miRNAs were prepared for further research (Table 1).

### Establishment of IRMS

**Figure 2:**
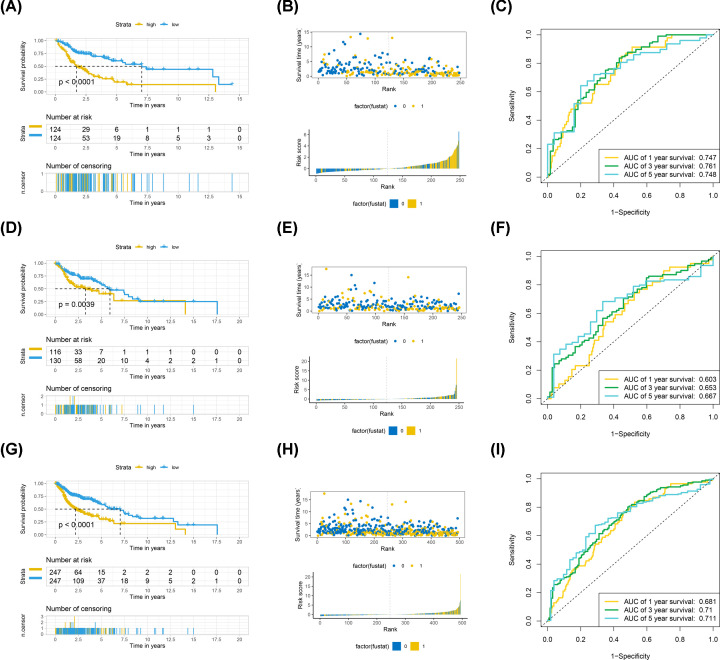
IRMS is a potent prognostic biomarker for OS in TCGA-HNSCC cohort The entire cohort was randomly divided into the training and testing cohorts at cut-off 5:5. (**A**) KM survival curves of OS according to IRMS groups in TCGA-HNSCC training cohort. (**B**) Bar-plot demonstrated that patients with high IRMS were more likely to be dead and patients with low IRMS were more inclined to alive in TCGA-HNSCC training cohort. Yellow indicated patients were dead and Blue indicated patients were alive. Dot-plot indicated that survival time of patients with high IRMS were less than patients with low IRMS in TCGA-HNSCC training cohort. Yellow indicated dead patients and Blue indicated living patients. (**C**) Time-dependent ROC curves of OS according to IRMS groups in TCGA-HNSCC training cohort. The AUC was assessed at 1, 3 and 5 years. (**D**) KM survival curves of OS according to IRMS groups in TCGA-HNSCC testing cohort. (**E**) Bar-plot demonstrated that patients with high IRMS were more likely to be dead and patients with low IRMS were more inclined to alive in TCGA-HNSCC testing cohort. Yellow indicated patients were dead and Blue indicated patients were alive. Dot-plot indicated that survival time of patients with high IRMS were less than patients with low IRMS in TCGA-HNSCC testing cohort. Yellow indicated dead patients and Blue indicated living patients. (**F**) Time-dependent ROC curves of OS according to IRMS groups in TCGA-HNSCC testing cohort. The AUC was assessed at 1, 3 and 5 years. (**G**) KM survival curves of OS according to IRMS groups in TCGA-HNSCC entire cohort. (**H**) Bar-plot demonstrated that patients with high IRMS were more likely to be dead and patients with low IRMS were more inclined to alive in TCGA-HNSCC entire cohort. Yellow indicated patients were dead and Blue indicated patients were alive. Dot-plot indicated that survival time of patients with high IRMS were less than patients with low IRMS in TCGA-HNSCC entire cohort. Yellow indicated dead patients and Blue indicated living patients. (**I**) Time-dependent ROC curves of OS according to IRMS groups in TCGA-HNSCC entire cohort. The AUC was assessed at 1, 3 and 5 years.

We randomly divided the entire TCGA-HNSCC cohort into training and testing cohorts at the cutoff 5:5. Then the LASSO and multivariate Cox regression analyses, which were responsible for dimension reduction, were used to establish an IRMS, which consisted of six miRNAs in training cohort (Supplementary Figure S1). KM survival curves and log-rank test indicated that miR-3127/miR-487b/miR-548k/miR-5690 was harmful, while miR-146a/miR-3913-2 was beneficial for patients with HNSCC (Supplementary Figure S2). The formula for IRMS risk-score was calculated as follows: expression of miR-146a * (−0.03975) + expression of miR-3127 * (0.02784) + expression of miR-3913-2 * (−0.02804) + expression of miR-487b * (0.01671) + expression of miR-548k * (0.01749) + expression of miR-5690 * (0.0477). After stratifying IRMS as the median cut-off, KM survival analysis demonstrated that IRMS low-risk group had a better OS than IRMS high-risk group in the training cohort (*P*<0.0001) (Figure 2A,B). Thus, time-dependent ROC analysis revealed that IRMS displayed an high accuracy of OS predicting in training cohort and AUC was 0.747 at 1 year, 0.761 at 3 years and 0.748 at 5 years (Figure 2C). Furthermore, we also validated the results in testing (*P*=0.0039) (Figure 2D,E) and entire cohort (*P*<0.0001) (Figure 2G,H) and found those were consistence with training cohort, indicating all the low-risk patients were associated with the better prognosis. The AUC with 1-, 3- and 5-years were 0.603, 0.653, 0.667 in testing cohort and 0.681, 0.71, 0.711 in the entire cohort, respectively (Figure 2F,I). All these indicated of a robust prediction value of IRMS.

### IRMS is highly correlated with the malignancy of HNSCC

**Figure 3:**
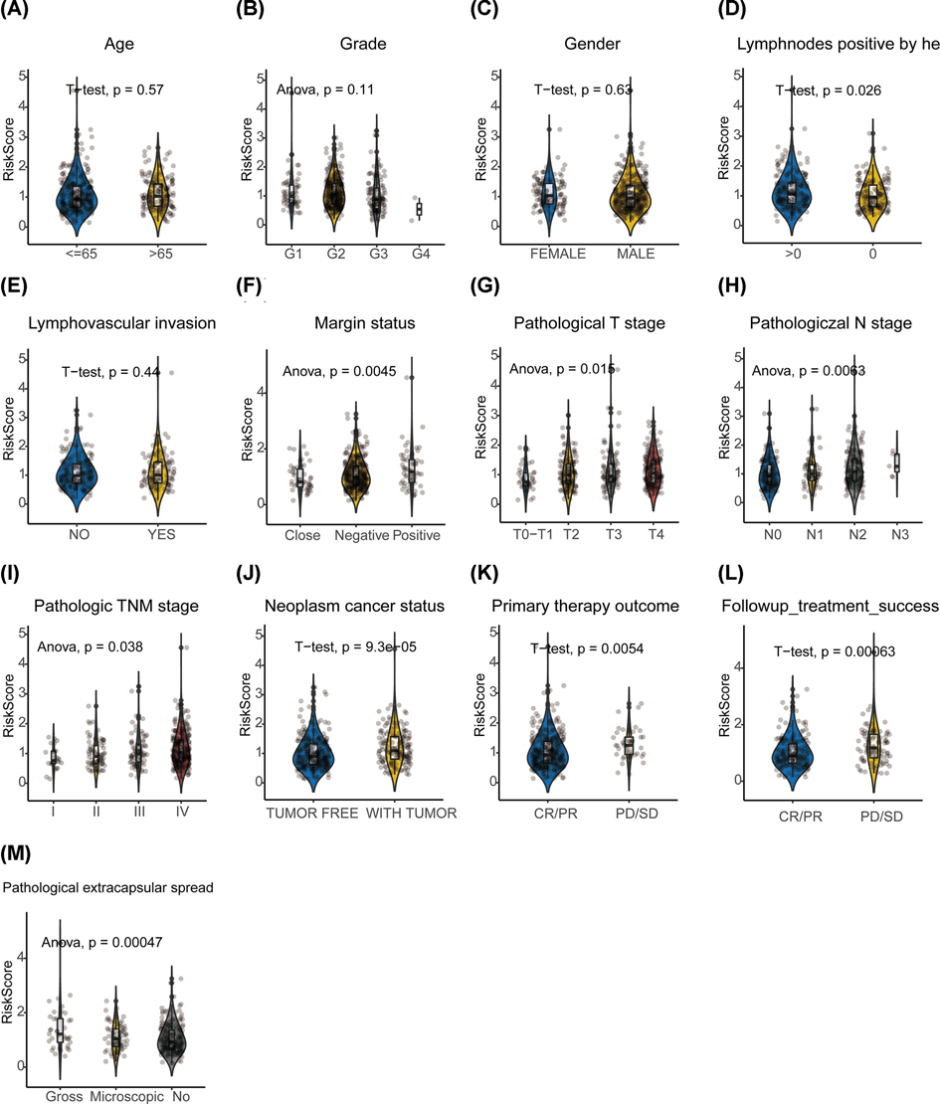
Association between the IRMS and clinicopathological characteristics Violin plots detected the correlation between IRMS risk-score and distinct subtypes of each clinicopathological characteristics through *t* test or one-way ANOVA. The stratified subtypes were listed as below: (**A**) Age: elder: >65 years, younger: ≤65 years. (**B**) Histological grade: G1, G2, G3 and G4. (**C**) Gender: female and male. (**D**) Lymph-nodes positive by HE: 0 and >0. (**E**) Lympho-vascular invasion status: NO and YES. (**F**) Margin statue: negative, close and positive. (**G**) Pathological T stage: T0–T1 stage, T2 stage, T3 stage and T4 stage. (**H**) Pathological N stage: N0 stage, N1 stage, N2 stage and N3 stage. (**I**) Pathological TNM stage: stage I, stage II, stage III and stage IV. (**J**) Neoplasm cancer status: tumour-free and with tumour. (**K**) Primary therapy outcome: CR/PR and PD/SD. (**L**) Follow-up treatment outcome: CR/PR and PD/SD. (**M**) Pathological extracapsular spread: none, microscopic and gross.

**Figure 4:**
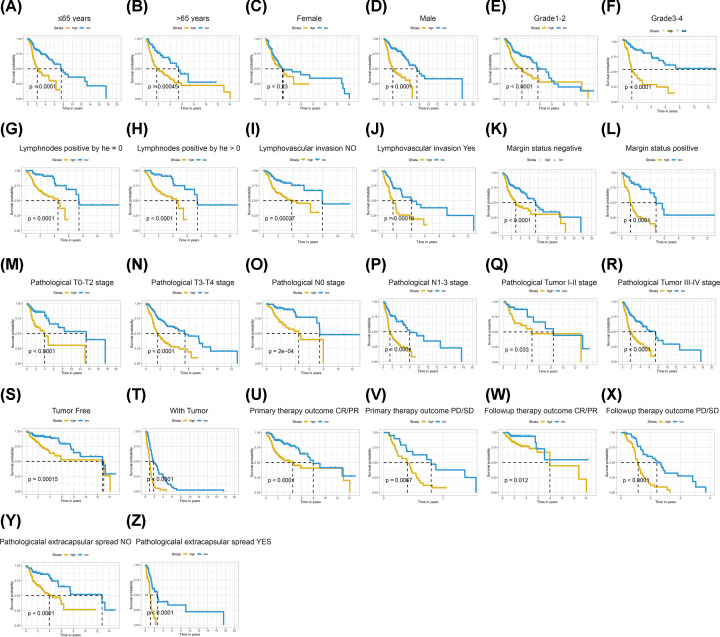
KM stratification survival analyses in TCGA-HNSCC cohort (**A**) Age ≤ 65 years. (**B**) Age > 65 years. (**C**) Female. (**D**) Male. (**E**) Grade: 1–2. (**F**) Grade; 3–4. (**G**) Lympho-nodes positive HE = 0. (**H**) Lympho-nodes positive by HE > 0. (**I**) Lympho-vascular invasion: NO. (**J**) Lympho-vascular invasion: YES. (**K**) Margin status: negative. (**L**) Margin status: positive. (**M**) Pathological T0–T2 stage. (**N**) Pathological T3–T4 stage. (**O**) Pathological N0 stage. (**P**) Pathological N1–3 stage. (**Q**) Pathological TNM I–II stage. (**R**) Pathological TNM III–IV stage. (**S**) Tumour-free. (**T**) With tumour. (**U**) Primary therapy outcome CR/PR. (**V**) Primary therapy outcome PD/SD. (**W**) Follow-up therapy outcome CR/PR. (**X**) Follow-up therapy outcome PD/SD. (**Y**) Pathological extracapsular spread: NO. (**Z**) Pathological extracapsular spread: YES.

The common clinical information of HNSCC, including age, gender, grade, lympho-nodes positive by HE, lympho-vascular invasion, margin status, pathological T stage, pathological N stage, pathological TNM stage, neoplasm cancer status, pathological extracapsular spread, primary therapy outcome and follow-up treatment outcome, were retrieved from TCGA-HNSCC cohort. KM survival curves showed that all above parameters were associated with the survival of patients with HNSCC despite gender and grade (Supplementary Figure S3). Then, we began investigating the relationship between IRMS and those clinical features. The violin plots revealed that IRMS was highly positively correlated with the lympho-nodes metastasis, margin status and pathological TNM stage and extracapsular spread. Moreover, the IRMS high-risk groups were more likely to be patients of with tumour, primary and follow-up treatment outcome with progressive disease/persistent disease/stable disease (PD/SD) (Figure 3). The *χ^2^* test demonstrated a similar result which was shown in cluster heat map (Figure 6A and Supplementary Table S1). Furthermore, we want to see whether our IRMS was independent with different clinicopathological characteristics. The stratification survival analyses indicated that IRMS was independent from all above variables and could make an efficient prediction of OS in almost all the subgroups (Figure 4).

### The IRMS was an independent prognostic factor in HNSCC

**Figure 5:**
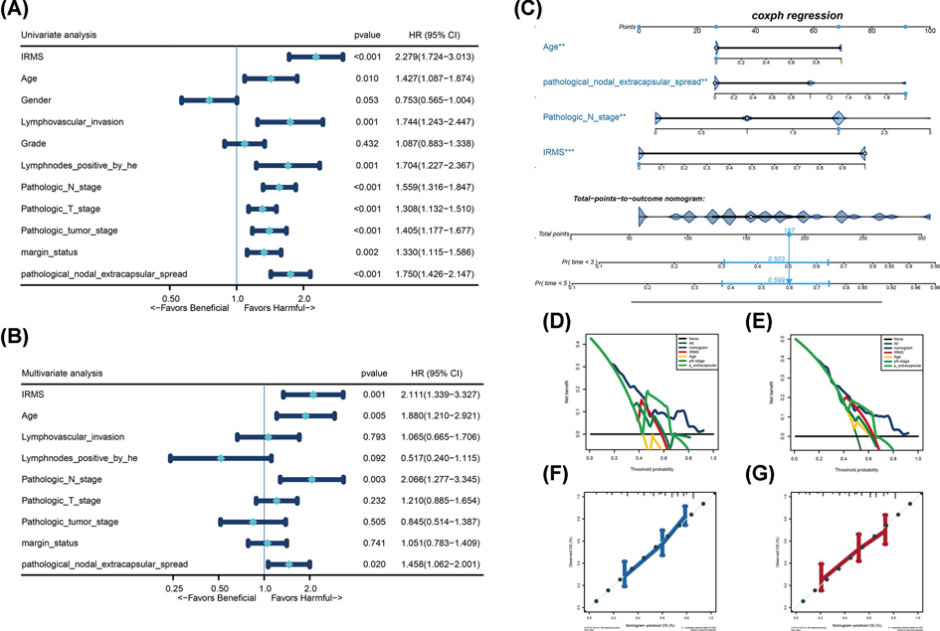
IRMS is an independent prognosis factor in HNSCC (**A,B**) Forest plot summary of IRMS and clinicopathological characteristics measured by the univariate and multivariable cox analyses. The *P*-value, HR and 95% confidence interval (CI) were indicated in the figure. (**C**) Nomograms which integrated with IRMS, age, pathological N stage and pathological extracapsular spread for predicting the probability of patient mortality at 3- or 5-year OS. (**D,E**) DCA curve of the nomograms and other variables in prediction of 3-year (D) and 5-year OS (E). (**F,G**) Calibration curves of the nomogram for predicting the OS at 3- (F), and 5-years (G) compared with the ideal model. The 45-degree line represents the ideal prediction. Abbreviation: HR, hazard ratio.

Although IRMS was significantly correlated with the malignancy and prognosis of the patients with HNSCC, we next want to figure out whether IRMS was an independent prognostic factor in HNSCC. By merging IRMS with clinicopathological characteristics mentioned above, univariate cox regression analysis showed that all parameters, except grade and gender, were harmful factors in HNSCC (Figure 5A). Through eliminating gender and grade, multivariate cox regression analysis indicated that age, IRMS, pathological extracapsular spread and pathological N stage were the only four independent prognostic factors responsible for OS predicting in patients with HNSCC (Figure 5B). Then a nomogram, which is quantitative scoring method, has been utilized to forecast the mortality of HNSCC patients by combination of the four independent prognostic factors (Figure 5C). Based on the established nomogram, each patient will get a total point by plus the points of four prognostic variables in the nomogram. We can see that if the patients get a higher total points, they were more likely to be dead at 3-years or 5-years, which demonstrated that the higher points the patients got meant the worse prognosis the HNSCC patients were. Moreover, the DCA curves demonstrated that our nomogram displayed a supreme advantage when compared with variables alone and could be of a high potential for clinical utility (Figure 5D,E). The calibration curves revealed that the nomogram have a highly similar prediction accuracy with the ideal model (Figure 5F,G).

### The immune infiltration landscape of HNSCC

**Figure 6:**
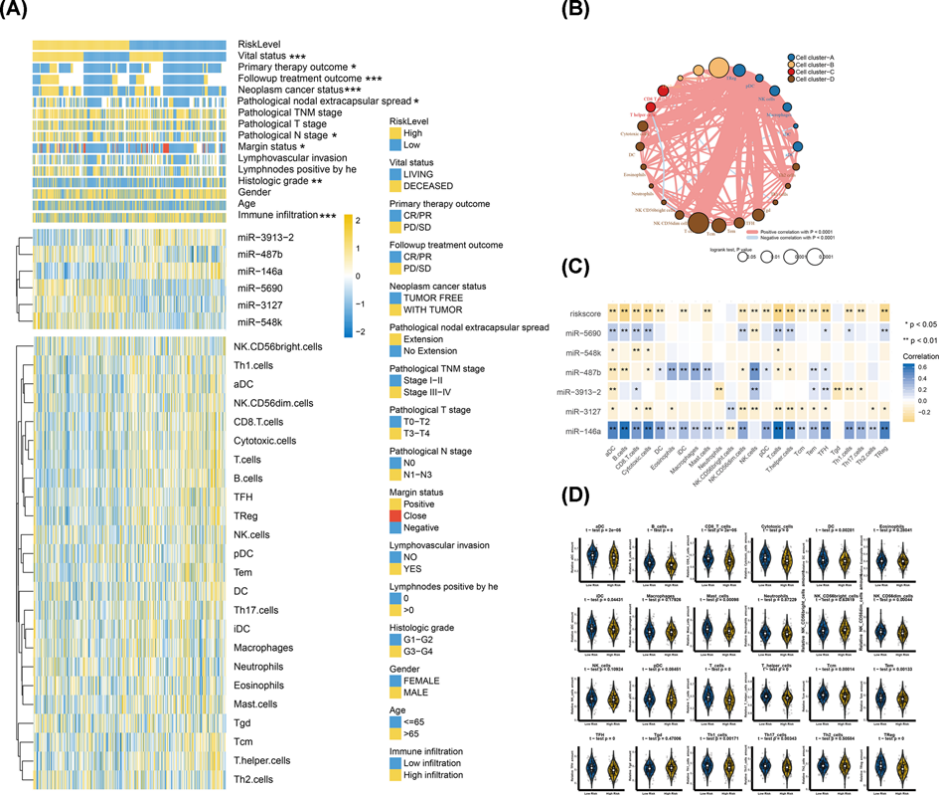
The IRMS is negatively correlated with immune infiltration (**A**) Cluster heat map showed the relative levels of immune-related miRNAs and 24 types of immune cells which were stratified by the IRMS risk-level in the TCGA-HNSCC cohort. Yellow meant levels of miRNAs and immune cells were up-regulated while blue meant down-regulated. The relationship between IRMS and indicated clinicopathological characteristics was measured with the *χ*^2^ test and shown in heat map. *P<0.05, **P<0.01, ***P<0.001. (**B**) Construction of immune cells network in TCGA-HNSCC cohort. The colour of cluster was listed as follows: Cell cluster (A), blue; Cell cluster (B), yellow; Cell cluster (C), red; Cell cluster (D), brown. The circle size represented log10 of *P*-value (Log-rank test *P*-value) of univariate cox regression analysis on OS for different immune cell types. The lines indicated that immune cells were connected with each other. And the thickness of the line meant the cellular interactions coefficient calculated by Spearman correlation analysis. Red meant positive correlation while blue meant negative correlation. (**C**) Correlation matrix showed the Spearman correlation between IRMS, immune-related miRNAs and 24 types of immune cells. The blue represented positive correlation and yellow indicated represented correlation. Shading color and asterisks indicated the correlation coefficients. *P<0.05, **P<0.01. (**D**) Violin plots demonstrated the association between IRMS risk-level and the amounts of 24 types of immune cells through *t* test.

In order to investigate whether IRMS could be a good representative of immune response status, its relationship with immune infiltration was studied. The ssGSEA was used to characterise the comprehensive landscape of immune infiltration in HNSCC. The relative amounts of each immune cell type in HNSCC cohort were listed in Supplementary Data S2. The KM survival curves revealed that almost half types of immune cells displayed beneficial effect on the prognosis of HNSCC patients (Supplementary Figure S4). Then we constructed an immune cells network to exhibit an overall view of cellular interaction, cellular clusters and prognosis on the OS of HNSCC patients (Figure 6B). There were four cell clusters within 24 immune cell types and the log-rank *P*-value as well as the correlation index could be found in Supplementary Data S3–S4. Furthermore, the cluster heat map indicated that IRMS low-risk group was involved with high immune infiltration compared with IRMS high-risk groups. Moreover, the Spearman correlation analyses showed that IRMS risk-score were significantly negatively correlated with levels of a majority of immune cell types, which exerted a robustly anti-tumour effect on HNSCC (Supplementary Data S5). Meanwhile, we also found that almost all the beneficial immune cells were filled in the IRMS low-risk group which indicated that immune-activate milieu might be the cause for its good prognosis (Figure 6C,D). Moreover, CIBERSORT algorithm was utilized to validate the results from the ssGSEA (Supplementary Data S6). We found that the distribution of immune cells was similar between two algorithms. Furthermore, the effector macrophages M1 was accumulated in IRMS low-risk group, while immunosuppressive macrophages M2 was concentrated in IRMS high-risk group.

### IRMS was associated with immune infiltraion and immune checkpoints

**Figure 7:**
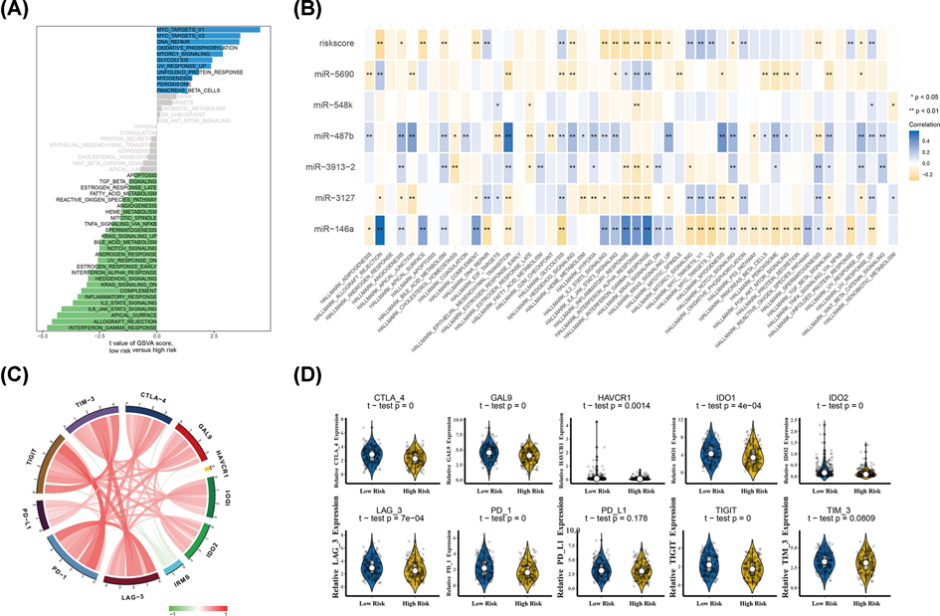
IRMS was involved in the regulation of immune response in HNSCC (**A**) The bar plot showed GSVA of hallmark gene sets in TCGA-HNSCC cohort. (**B**) Correlation matrix of IRMS risk-score, immune-related miRNAs and the relative levels of hallmark gene sets. (**C**) The correlation chord chart between IRMS and immune checkpoint-relevant genes, including CTLA-4, GAL9, HAVCR1, IDO-1, IDO-2, LAG-3, PD-1, PD-L1, TIGHT and TIM-3. (**D**) Violin plots showed the correlation between IRMS and above immune checkpoint-relevant genes. Abbreviations: CTLA-4, cytotoxic T lymphocyte-associated protein 4; GAL9, LGALS9.

As negatively associated with the immune infiltration, we next utilized GSVA to make clear dynamics of biological processes and pathways based on the Hallmark gene sets which was stratified by the IRMS risk-level. The detailed information of GSVA was listed in Supplementary Data S7. The results showed that allograft rejection, complement, IL2-STAT5 signalling, IL6-JAK-STAT3 signalling, inflammatory response, IFNα response and IFNγ response, which were representatives of immune activation, were enriched in IRMS low-risk group, while the IRMS high-risk group was enriched in MYC target and glycolysis pathway that played a vital role in tumorigenesis (Figure 7A and Supplementary Data S7). Moreover, we also found that the immune activation-related pathways mentioned above were significantly negatively correlated with the IRMS risk-score (Figure 7B and Supplementary Data S8).

Recently the expression of immune checkpoints was emerged as predictive biomarkers for immunotherapy in multiple malignancies. Due to immune-activated atmosphere in IRMS low-risk patients, we next wanted to know whether there existed a correlation between IRMS and immune checkpoints. Thus, the common immune-checkpoint-related candidate genes, including CD274 (PD-L1), cytotoxic T lymphocyte-associated protein 4 (CTLA-4), LAG-3, LGALS9 (GAL9), HAVCR, HAVCR2 (TIM-3), IDO1, IDO2, PDCD1 (PD-1) and TIGHT, were enrolled to assess the relationship with IRMS. Then we found that all immune checkpoints were positive correlated with each other, while IRMS risk-score was significantly negatively correlated to the expression of them (Figure 7C). In addition, we investigated the expression of immune checkpoints between the IRMS low/high-risk HNSCC patients. The results showed that the expression of CTLA-4, GAL9, HAVCR1, IDO-1, IDO-2, LAG-3, PD-1 and TIGHT in the IRMS low-risk group was significantly higher than that in the high-risk HNSCC group (*P*<0.05), indicating that the activation of efficiency immune infiltration might be triggered by up-regulation of the immune checkpoints (Figure 7D).

## Discussion

Nowadays we have seen the development of the next-generation sequencing and bioinformatics, as well as the release of many public databases such as Gene Expression Omnibus (GEO) and TCGA. Based on this background, analysis and assessment of the genome and transcriptome sequencing data in different types of cancers or pan-cancer would give us a comprehensive understanding of tumorigenesis which is triggered by genomic and epigenetic dysfunctions [40]. As public data are easy to obtain, many ncRNAs such as miRNAs, long ncRNAs (lncRNAs), circular RNAs (circRNAs) etc., which are thought to be ‘junk’ at first, are found to play critical roles in tumorigenesis.

As one of the most studied ncRNAs, miRNAs have a unique nature that a single miRNA could regulate a lot of RNA transcripts, and be regulated by others at the same time [41]. Therefore, aberrant expression of miRNAs could disrupt the tightly controlled RNA networks, which has been shown to initiate and promote many human diseases, including cancers [42]. Thus, miRNAs were found to be oncogenes or tumour suppressors which was deeply involved in various biological processes, such as cell proliferation, invasion, apoptosis, metastasis and drug resistance [22,43]. A lot of miRNAs, including miR-10a, miR-27b and miR-33a etc., were found to be associated with non-small cell lung cancer (NSCLC) progression, metastasis and invasion [44]. MiR-21 was found to be strikingly higher in serum of patients with hormone-refractory prostate cancer who were resistant to docetaxel-based chemotherapy, indicating its role in regulating drug-resistance [45]. Summerer et al. reported that circulating miR-142-3p, miR-186-5p, miR-195-5p, miR-374b-5p and miR-574-3p were up-regulated in the plasma of HNSCC patients and were also correlated with poor prognosis in HNSCC [46]. Moreover, researchers have also found alteration of miRNAs could be influenced by priming of innate and adaptive immune response, such as T-cell development, differentiation. Conversely, more and more miRNAs were reported to regulate tumour immune microenvironment (TIME) such as initiation and maintaining inflammatory mediator production and immunosuppressive milieu formation [47]. With the help of bioinformatics and machinery methods, many studies have utilized transcriptomic data to construct the miRNAs signature for predicting the prognosis of multiple cancers, including prostate cancer [48], breast cancer [49], as well as HNSCC [50]. Furthermore, some even focused on investigating the IRMS in breast cancer [51] etc., but they were not explored in HNSCC yet.

As HNSCC is recognised as immunogenic cancer, we aimed to find some immune-related miRNAs and establish a signature which could be representative of the immune response status. At first, we have utilized the gene sets the M13664 (immune system process) and M19817 (immune response) to represent immune response in TCGA-HNSCC cohort. With the strict criterion mentioned above, we got 11 prognostic immune-related miRNAs through ssGSEA and univariate cox regression analysis. By using LASSO and multivariate cox regression analyses, we have constructed a IRMS, including miR-146a, miR-3127, miR-3913-2, miR-487b, miR-548k and miR-5690. IRMS was capable of predicting OS and highly associated with the malignancy of HNSCC. Although HNSCC was also a heterogeneous disease, the results from the stratification analyses indicated that IRMS was independent from all of them and be able to predict prognosis in all subgroups of the clinical features. In addition, univariate and multivariate cox regression analyses, both demonstrated that IRMS was an independent prognostic factor when combination with other variable such as pathological N stage, etc. Moreover, after construction of the nomogram with selected independent prognostic factors, we found IRMS performed well in survival prediction and contributed more within this model. All of these indicated that IRMS was associated with oncogenic role and was potent biomarker which could predict prognosis in HNSCC.

Recently pre-treatment immune infiltration status has been identified as vital invaluable predictor for forecasting the prognosis and immunotherapy response in a variety of clinical trials with ICIs [52,53]. As IRMS was a signature-related immune response, its relationship with immune infiltration was studied. A comprehensive analysis of immune infiltration landscape was estimated via ssGSEA and CIBERSORT algorithms to calculate the amount of immune cells in TCGA-HNSCC cohort. Amazingly, ssGSEA demonstrated that IRMS low-risk group was highly infiltrated with almost all immune effector cells, such as B cell, CD8^+^ T cells and cytotoxic cells etc., which could initiate recognition process and lead to the eradication of the tumour cells [13,54]. Furthermore, the results from CIBERSORT demonstrated a similar distribution of immune cells with ssGSEA. Moreover, the effector macrophages M1 and immunosuppressive macrophages M2 was concentrated in IRMS low- and high-risk groups, respectively, which demonstrated immune-activation atmosphere do exist in IRMS low-risk group and occurrence of immunosuppressive atmosphere in IRMS high-risk group. But we also found that immunosuppressive cells like NK CD56dim cells and Treg cells are also up-regulated in IRMS low-risk group, which seemed to be going in the opposite direction. However, when clearly noticing the immune cells network, we were surprised to find that Treg and NK CD56dim cells were strongly positively correlated with all immune cells, no matter harmful or beneficial. This might be caused by the existence of a negative feedback loop that immunosuppressive cells could respond to the alteration of other immune cells and attempt to hamper the eliminating of tumour cells in HNSCC. In this context, we think the good prognosis of the IRMS low-risk patients might be owing to a large amount of immune effector cells *in situ* within TIME.

Interestingly, the results from the function annotation analysis also demonstrated that the enrichment of immune response related pathways, such as complement, IL2-STAT5 signalling, IL6-JAK-STAT3 signalling, inflammatory response, IFNα response and IFNγ response, which represent immune activation, were activated in IRMS low-risk group, while the IRMS high-risk group were enriched in the oncogenic pathways such as MYC target and glycolysis. The results from the immune infiltration landscape and function annotation were amazingly consistent, which suggested that our IRMS was a good representative of immune response status in HNSCC. The immune checkpoints, such as CTLA-4, PD-1/PD-L1 etc. were reported to be responsible for the immunotherapy response in many cancers [55]. Next we want to figure out the relationship between IRMS and immune checkpoints. Thus, we found that the expression of immune checkpoints all increased in IRMS low-risk group, indicating that the infiltration of immune effector cells might be induced by up-regulation of the immune checkpoints.

## Conclusion

We have made a comprehensive analysis of immune infiltration landscape and established a prognostic and predictive IRMS for representative of immune response in HNSCC, which has opened our view in immunotherapies and may provide a useful scoring system for clinical utility.

## Supplementary Material

Supplementary Figures S1-S5 and Table S1

Supplementary Data S1-S7

## Data Availability

All data generated or analysed during the present study are included in this published article and its supplementary file information files.

## Abbreviations

AUC: area under curve
CTLA-4: cytotoxic T lymphocyte-associated protein 4
DC: dendritic cell
DCA: decision curve analysis
FPKM: Fragments Per Kilobase per Million
GAL9: LGALS9
GEO: Gene Expression Omnibus
GSVA: Gene Set Variation Analysis
HE: Hematoxylin and Eosin
HNSCC: head and neck squamous cell carcinoma
HR: hazard ratio
HTSeq: HT-Sequencing
ICI: immune-checkpoint inhibitor
IFN: interferon
IRMS: immune-related miRNAs signature
KM: Kaplan–Meier
LASSO: least absolute shrinkage and selection operator
miRNA: microRNA
MSigDB: Molecular Signatures Database
ncRNA: non-coding RNA
NK: natural killer cell
OS: overall survival
PD: progressive disease/persistent disease
PD-1: programmed death-1
PD-L1: programmed death-ligand-1
ROC: receiver operating characteristic curve
RPM: reads per million miRNAs
SD: stable disease
ssGSEA: single-sample gene set enrichment analysis
TCGA: The Cancer Genome Atlas
TIME: tumour immune microenvironment
TNM: tumour node metastasis
TPM: transcripts per million reads
Treg: regulatory T cell
UCSC: University of California, Santa Cruz
