# Supplementary material for: Immune-related miRNA signature identifies prognosis and immune landscape in head and neck squamous cell carcinomas
Source: Biosci Rep. 2020 Nov 17;40(11):BSR20201820. doi: 10.1042/BSR20201820 (PMC7670576; doi:10.1042/BSR20201820)
Supplement: Supplementary Figures S1-S5 and Table S1 [file BSR-2020-1820_supp.pdf]

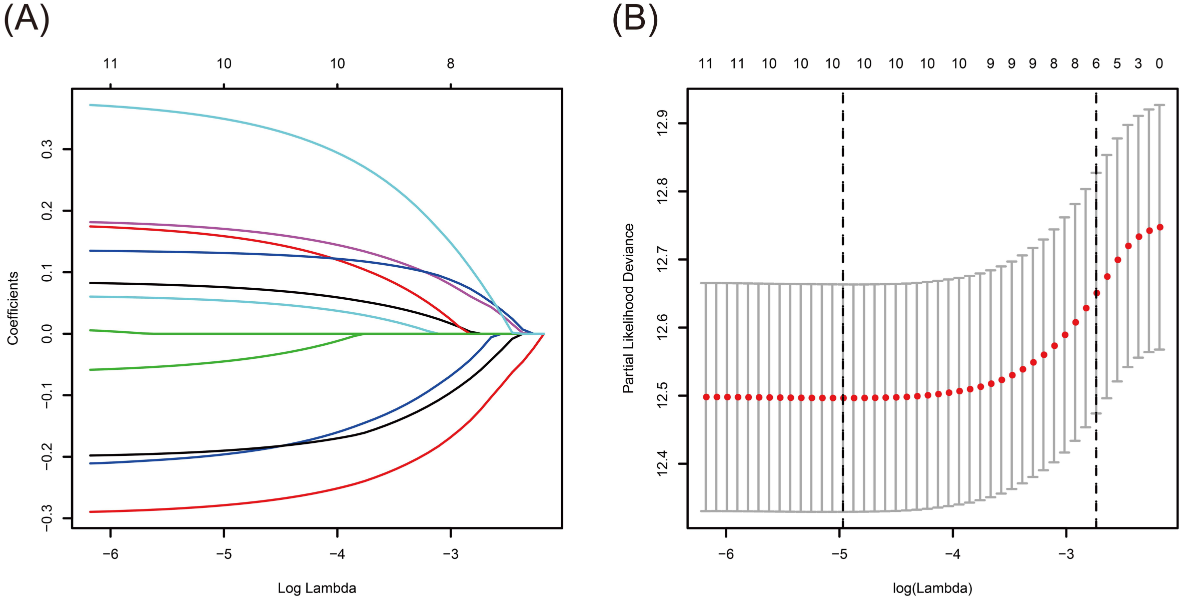
(A)
(B)
11
10
10
8
Coefficients
0.3
0.2
0.1
0.0
−0.1
−0.2
−0.3
−6
−5
−4
−3
Log Lambda
11 11 10 10 10 10 10 10 10 9 9 9 8 8 6 5 3 0
Partial Likelihood Deviance
12.9
12.8
12.7
12.6
12.5
12.4
log(Lambda)

Figure S1. Construction of prognostic immune-related miRNAs signature (IRMS) through LASSO Cox regression model.(A) The LASSO coefficients profiles of 11prognostic and immune-related candidate miRNAs. (B) Tuning parameter (λ) selection cross - validation error curve. The optimal values of the minimum criteria and the 1 - SE criteriawere drawn with the vertical dashed lines. The 1 - SE criteria were selected for establishingIRLS.

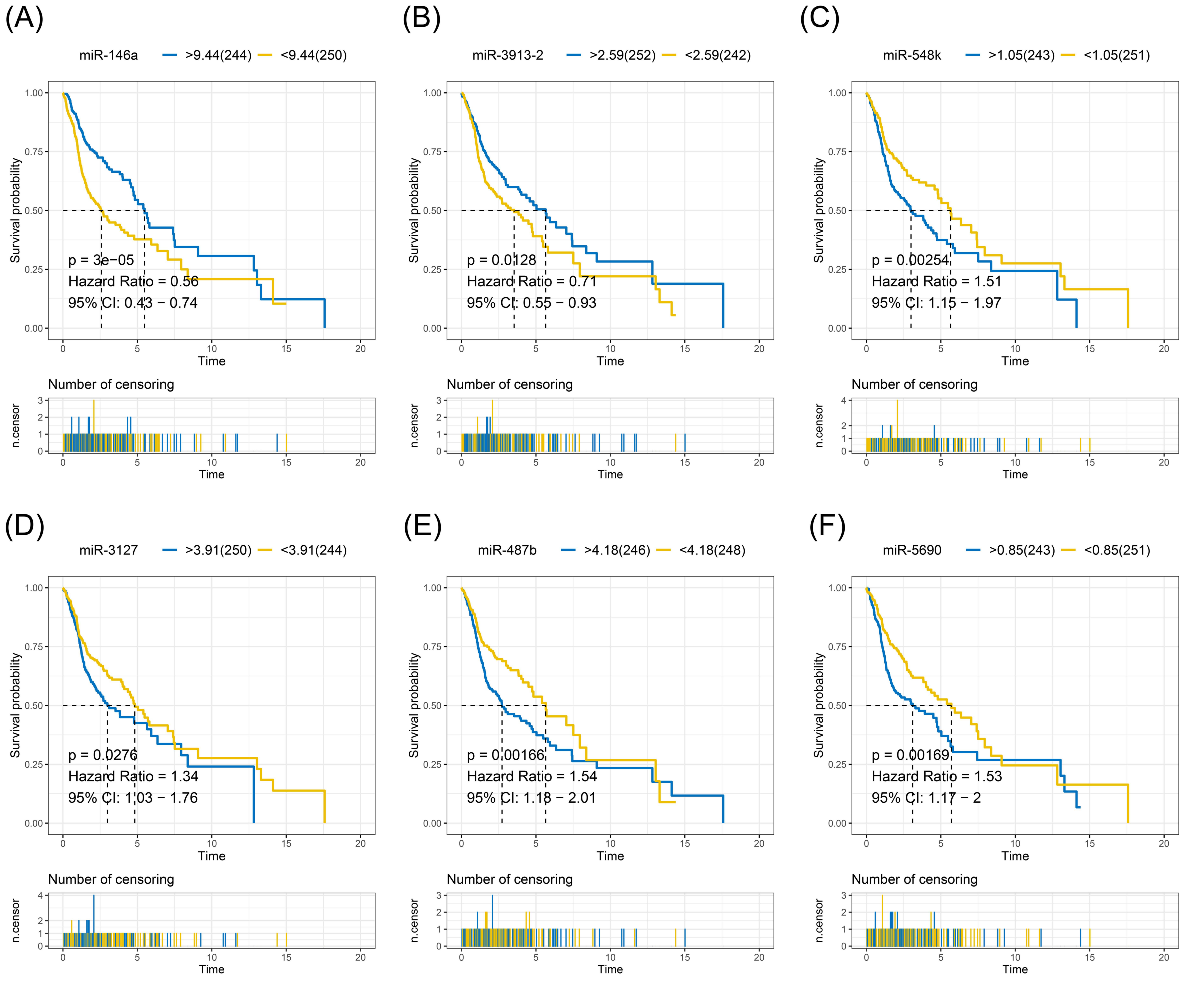

(A)
miR-146a >9.44(244) <9.44(250)
p = 3e−05
Hazard Ratio = 0.56
95% CI: 0.43 − 0.74
(B)
miR-3913-2 >2.59(252) <2.59(242)
p = 0.0128
Hazard Ratio = 0.71
95% CI: 0.55 − 0.93
(C)
miR-548k >1.05(243) <1.05(251)
p = 0.00254
Hazard Ratio = 1.51
95% CI: 1.15 − 1.97
(D)
miR-3127 >3.91(250) <3.91(244)
p = 0.0276
Hazard Ratio = 1.34
95% CI: 1.03 − 1.76
(E)
miR-487b >4.18(246) <4.18(248)
p = 0.00166
Hazard Ratio = 1.54
95% CI: 1.18 − 2.01
(F)
miR-5690 >0.85(243) <0.85(251)
p = 0.00169
Hazard Ratio = 1.53
95% CI: 1.17 − 2
Survival probability
Time
Number of censoring
n.censor

Figure S2. Kaplan-Meier survival analysis and log-rank test of6 immune-related miRNA, includingmiR-146a (A), miR-3913-2 (B), miR-548k (C),miR-3127 (D),miR-487b (E),and miR-5690 (F).

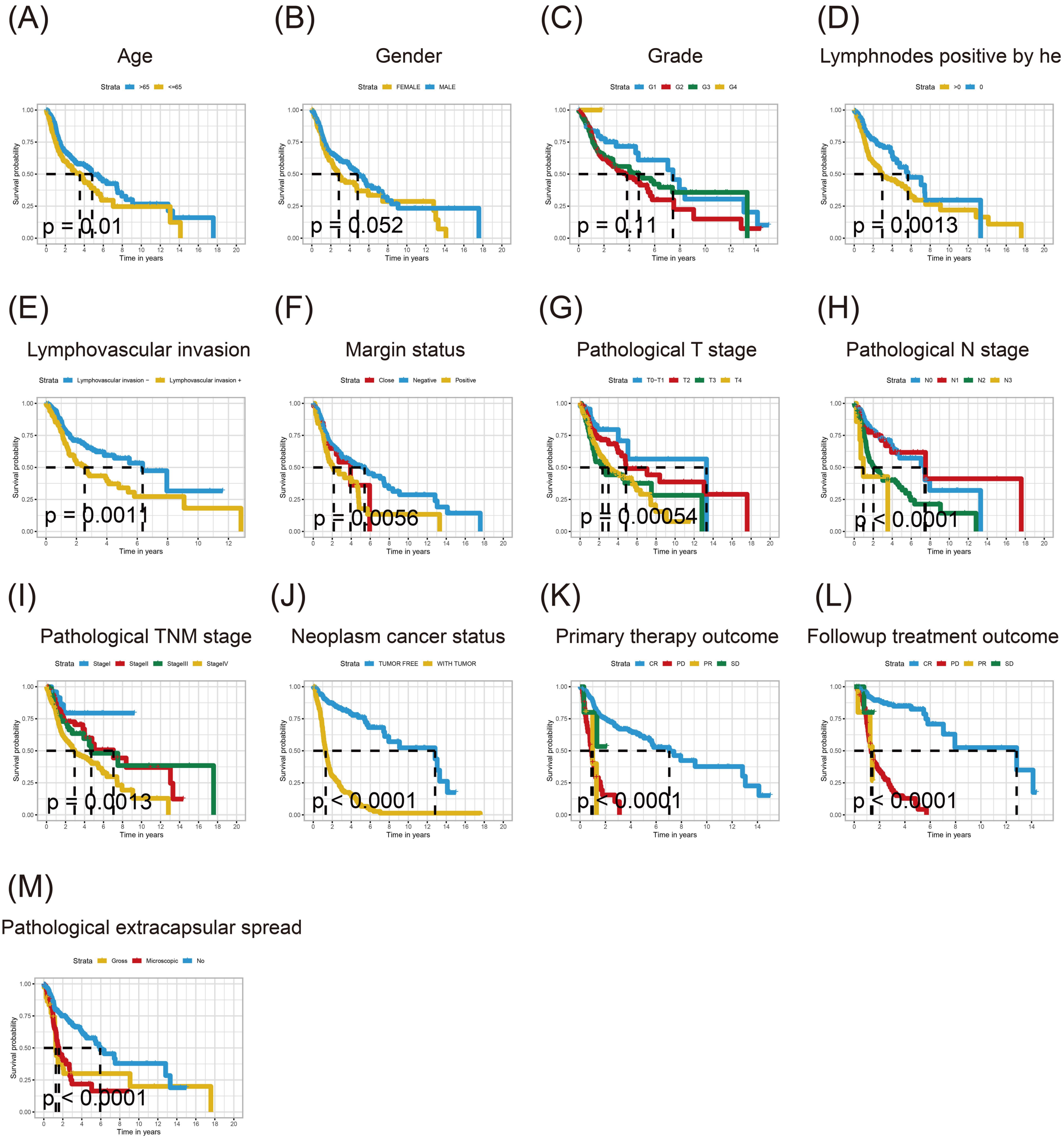
(A)
Age
Strata >65 <=65
p = 0.01
(B)
Gender
Strata FEMALE MALE
p = 0.052
(C)
Grade
Strata G1 G2 G3 G4
p = 0.11
(D)
Lymphnodes positive by he
Strata >0 0
p = 0.0013
(E)
Lymphovascular invasion
Strata Lymphovascular invasion − Lymphovascular invasion +
p = 0.0011
(F)
Margin status
Strata Close Negative Positive
p = 0.0056
(G)
Pathological T stage
Strata T0−T1 T2 T3 T4
p = 0.00054
(H)
Pathological N stage
Strata N0 N1 N2 N3
p < 0.0001
(I)
Pathological TNM stage
Strata StageI StageII StageIII StageIV
p = 0.0013
(J)
Neoplasm cancer status
Strata TUMOR FREE WITH TUMOR
p < 0.0001
(K)
Primary therapy outcome
Strata CR PD PR SD
p < 0.0001
(L)
Followup treatment outcome
Strata CR PD PR SD
p < 0.0001
(M)
Pathological extracapsular spread
Strata Gross Microscopic No
p < 0.0001
Survival probability
Time in years

Figure S3. Kaplan-Meiersurvival analysis of differentclinicopathological characteristics, including age (A),gender (B),grade (C),lymphonodes positive by hematoxylin and eosin (HE) (D), lymphovascular invasion status (E), margin status (F), pathological T stage (G), pathological N stage (H), pathological tumour stage (I), neoplasm cancer status (J), pathological extracapsular spread (K), primary therapy outcome (L) and follow-up treatment outcome (M).

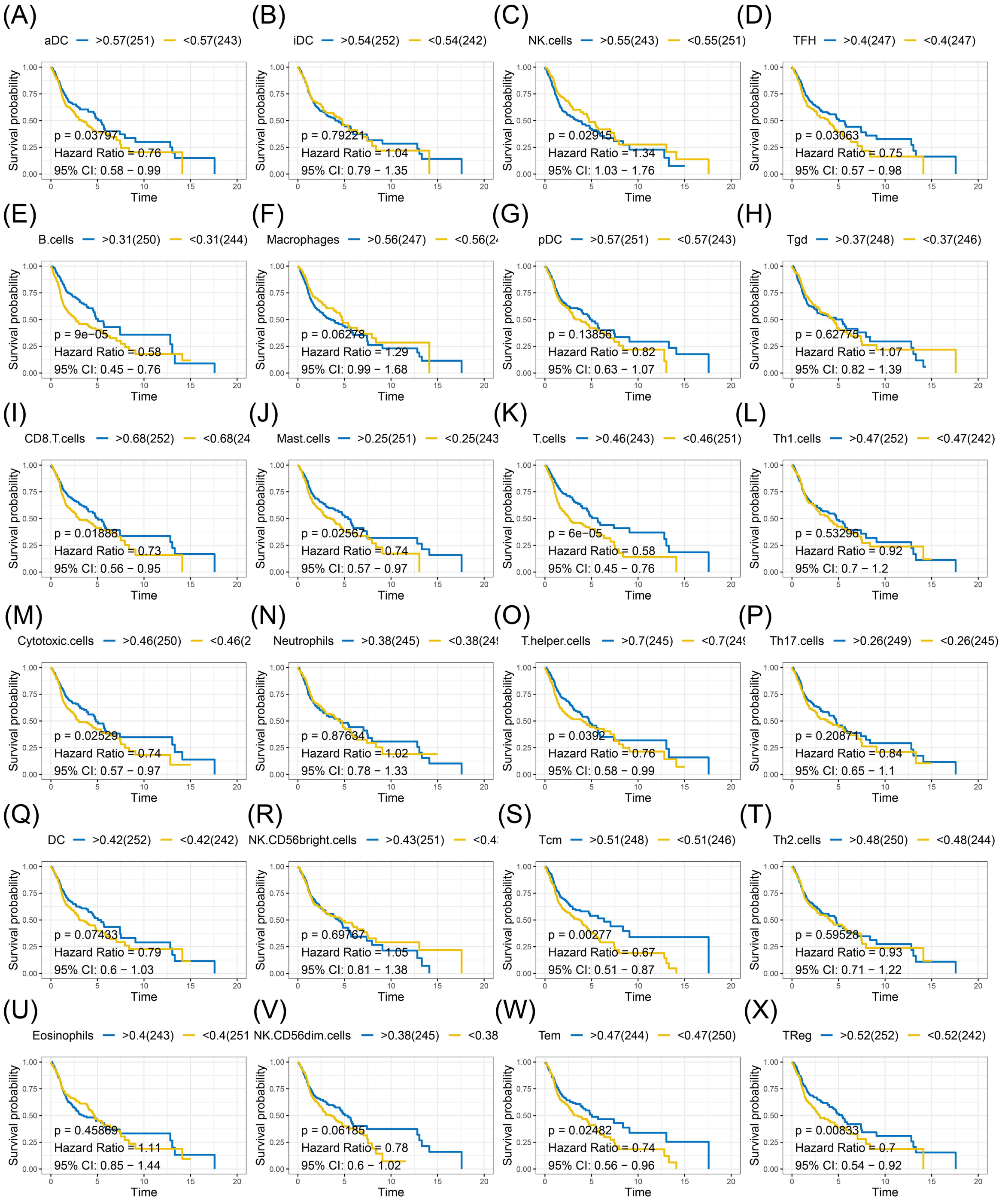

(A)
aDC >0.57(251) <0.57(243)
p = 0.03797
Hazard Ratio = 0.76
95% CI: 0.58 − 0.99
(B)
iDC >0.54(252) <0.54(242)
p = 0.79221
Hazard Ratio = 1.04
95% CI: 0.79 − 1.35
(C)
NK.cells >0.55(243) <0.55(251)
p = 0.02915
Hazard Ratio = 1.34
95% CI: 1.03 − 1.76
(D)
TFH >0.4(247) <0.4(247)
p = 0.03063
Hazard Ratio = 0.75
95% CI: 0.57 − 0.98
(E)
B.cells >0.31(250) <0.31(244)
p = 9e−05
Hazard Ratio = 0.58
95% CI: 0.45 − 0.76
(F)
Macrophages >0.56(247) <0.56(2
p = 0.06278
Hazard Ratio = 1.29
95% CI: 0.99 − 1.68
(G)
pDC >0.57(251) <0.57(243)
p = 0.13856
Hazard Ratio = 0.82
95% CI: 0.63 − 1.07
(H)
Tgd >0.37(248) <0.37(246)
p = 0.62775
Hazard Ratio = 1.07
95% CI: 0.82 − 1.39
(I)
CD8.T.cells >0.68(252) <0.68(24
p = 0.01888
Hazard Ratio = 0.73
95% CI: 0.56 − 0.95
(J)
Mast.cells >0.25(251) <0.25(243
p = 0.02567
Hazard Ratio = 0.74
95% CI: 0.57 − 0.97
(K)
T.cells >0.46(243) <0.46(251)
p = 6e−05
Hazard Ratio = 0.58
95% CI: 0.45 − 0.76
(L)
Th1.cells >0.47(252) <0.47(242)
p = 0.53296
Hazard Ratio = 0.92
95% CI: 0.7 − 1.2
(M)
Cytotoxic.cells >0.46(250) <0.46(2
p = 0.02529
Hazard Ratio = 0.74
95% CI: 0.57 − 0.97
(N)
Neutrophils >0.38(245) <0.38(24
p = 0.87634
Hazard Ratio = 1.02
95% CI: 0.78 − 1.33
(O)
T.helper.cells >0.7(245) <0.7(24
p = 0.0392
Hazard Ratio = 0.76
95% CI: 0.58 − 0.99
(P)
Th17.cells >0.26(249) <0.26(245)
p = 0.20871
Hazard Ratio = 0.84
95% CI: 0.65 − 1.1
(Q)
DC >0.42(252) <0.42(242)
p = 0.07433
Hazard Ratio = 0.79
95% CI: 0.6 − 1.03
(R)
NK.CD56bright.cells >0.43(251) <0.4
p = 0.69767
Hazard Ratio = 1.05
95% CI: 0.81 − 1.38
(S)
Tcm >0.51(248) <0.51(246)
p = 0.00277
Hazard Ratio = 0.67
95% CI: 0.51 − 0.87
(T)
Th2.cells >0.48(250) <0.48(244)
p = 0.59528
Hazard Ratio = 0.93
95% CI: 0.71 − 1.22
(U)
Eosinophils >0.4(243) <0.4(251
p = 0.45869
Hazard Ratio = 1.11
95% CI: 0.85 − 1.44
(V)
NK.CD56dim.cells >0.38(245) <0.38
p = 0.06185
Hazard Ratio = 0.78
95% CI: 0.6 − 1.02
(W)
Tem >0.47(244) <0.47(250)
p = 0.02482
Hazard Ratio = 0.74
95% CI: 0.56 − 0.96
(X)
TReg >0.52(252) <0.52(242)
p = 0.00833
Hazard Ratio = 0.7
95% CI: 0.54 − 0.92
Survival probability
Time

Figure S4. Kaplan-Meier survival analyses of each immune cell types in TCGA-HNSCC cohort.

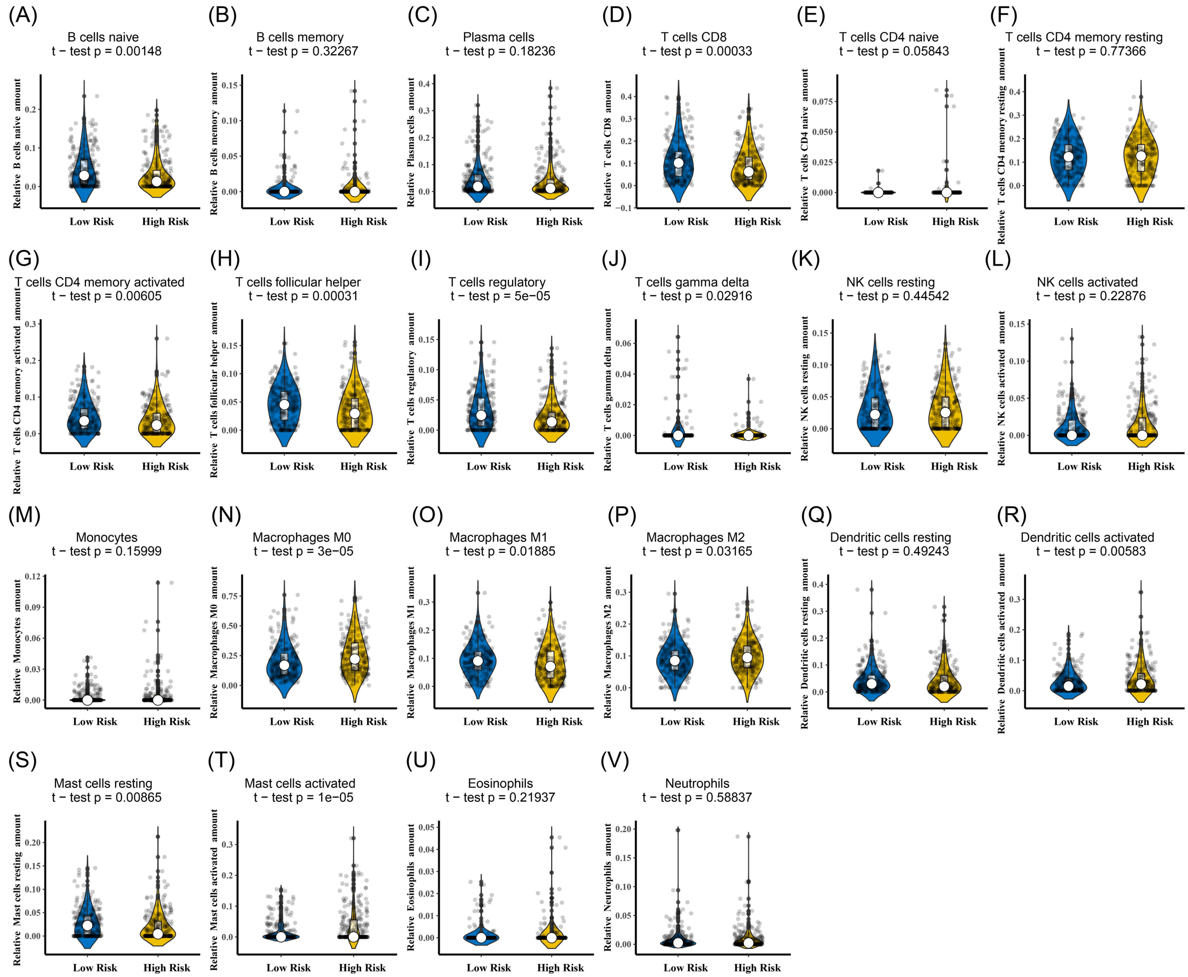

(A)
B cells naive
t − test p = 0.00148
Relative B cells naive amount
Low Risk
High Risk
(B)
B cells memory
t − test p = 0.32267
Relative B cells memory amount
(C)
Plasma cells
t − test p = 0.18236
Relative Plasma cells amount
(D)
T cells CD8
t − test p = 0.00033
Relative T cells CD8 amount
(E)
T cells CD4 naive
t − test p = 0.05843
Relative T cells CD4 naive amount
(F)
T cells CD4 memory resting
t − test p = 0.77366
Relative T cells CD4 memory resting amount
(G)
T cells CD4 memory activated
t − test p = 0.00605
Relative T cells CD4 memory activated amount
(H)
T cells follicular helper
t − test p = 0.00031
Relative T cells follicular helper amount
(I)
T cells regulatory
t − test p = 5e−05
Relative T cells regulatory amount
(J)
T cells gamma delta
t − test p = 0.02916
Relative T cells gamma delta amount
(K)
NK cells resting
t − test p = 0.44542
Relative NK cells resting amount
(L)
NK cells activated
t − test p = 0.22876
Relative NK cells activated amount
(M)
Monocytes
t − test p = 0.15999
Relative Monocytes amount
(N)
Macrophages M0
t − test p = 3e−05
Relative Macrophages M0 amount
(O)
Macrophages M1
t − test p = 0.01885
Relative Macrophages M1 amount
(P)
Macrophages M2
t − test p = 0.03165
Relative Macrophages M2 amount
(Q)
Dendritic cells resting
t − test p = 0.49243
Relative Dendritic cells resting amount
(R)
Dendritic cells activated
t − test p = 0.00583
Relative Dendritic cells activated amount
(S)
Mast cells resting
t − test p = 0.00865
Relative Mast cells resting amount
(T)
Mast cells activated
t − test p = 1e−05
Relative Mast cells activated amount
(U)
Eosinophils
t − test p = 0.21937
Relative Eosinophils amount
(V)
Neutrophils
t − test p = 0.58837
Relative Neutrophils amount

Figure S5. **The immune infiltration landscape calculated with CIBERSORT algorithm in TCGA-HNSCC cohort**. (A-V) Violin plots demonstrated the association between IRMS risk-score level and the levels amounts of 24 types of immune cell through t-test.

Supplementary Table S1. χ^2test forall patients with TCGA-HNSCCcohort according to the IRMS stratified by clinicopathologicalcharacteristics

Clinicopathological characters	p value
Age	0.187847398
followup treatment success	0.000399333
Gender	0.360143363
Grade	0.007205319
Immune infiltration	2.36E-05
Lymphnodes positive by he	0.176165457
Lymphovascular invasion	0.882387491
margin status	0.020139077
Pathological N stage	0.028809104
Pathological T stage	0.369791253
Pathological tumor stage	0.086788563
pathological nodal extracapsular spread	0.037585433
person neoplasm cancer status	0.000568723
primary therapy outcome success	0.022692328
vital status	9.61E-05
