# Supplementary material for: Immune-related miRNA signature identifies prognosis and immune landscape in head and neck squamous cell carcinomas
Source: Biosci Rep. 2020 Nov 17;40(11):BSR20201820. doi: 10.1042/BSR20201820 (PMC7670576; doi:10.1042/BSR20201820)
Supplement: Supplementary Data S1-S7 [file BSR-2020-1820_supp1.zip › BSR-2020-1820_suppSD.doc]

**Table S1. Summary ofrelative levels of 24 types of immunecells based on ssGSEA in TCGA-HNSCC cohort**.

**Table S2. Summary of difference in survival rate for immune cells network in TCGA-HNSCC cohort.**

**Table S3. Summary of spearman correlation for immune cells network in TCGA-HNSCC cohort.**

**Table S4. Summary of correlation matrix for IRMS, immune-related miRNAs and immune-related miRNAs in TCGA-HNSCC cohort.**

**Table S5. Summary of relative levels of 22 types of immune cells based on CIBERSORT algorithm in TCGA-HNSCC cohort.**

**Table S6. Summary of GSVA for hallmark gene sets in TCGA-HNSCC cohort.**

**Table S7. Summary of correlation matrix for IRMS and hallmark gene setsin TCGA-HNSCC cohort.**
